# Deviation of Spatial Representation and Asymmetric Saccadic Reaction Time in Hemi-Parkinson’s Disease

**DOI:** 10.3389/fnagi.2018.00084

**Published:** 2018-03-28

**Authors:** Dongfang Shen, Min Li, Ying Zhou, Lixin Liang, Lu Zhang, Wangzikang Zhang, Mingsha Zhang, Yujun Pan

**Affiliations:** ^1^Department of Neurology, The First Clinical College of Harbin Medical University, Harbin, China; ^2^Department of Neurology, The Fourth Clinical College of Harbin Medical University, Harbin, China; ^3^State Key Laboratory of Cognitive Neuroscience and Learning, Beijing Normal University, Beijing, China; ^4^Department of Critical Care Medicine, Beijing Tiantan Hospital, Capital Medical University, Beijing, China; ^5^Department of Neuroscience, Columbia University, New York, NY, United States

**Keywords:** egocentric reference frame, eye position, asymmetric spatial perception, subjective straight ahead, saccadic reaction time, Parkinson’s disease

## Abstract

**Background**: Patients with Parkinson’s disease (PD) commonly show spatially asymmetric behaviors, such as veering while attempting to walk in a straight line. While there is general agreement that the lateral motor dysfunction contributes to asymmetric behaviors in PD, it is dispute regarding whether the spatial perception is also biased. In addition, it is not clear whether PD impairs the speed of spatial information process, i.e., the efficiency of information process.

**Objectives**: To assess the visuospatial representation and efficiency of spatial information processing in hemi-PD.

**Methods**: Two saccadic tasks were employed: non-spatial cue evoked saccade and spatial cue evoked saccade. In the former task, an identical visual stimulus (appeared on the body mid-sagittal plane) was artificially associated with a fixed saccadic target (left or right) in a given session. In the latter task, subjects were instructed to make a rightward or leftward saccade based on the perceived location of a visual cue (left vs. right side of the body mid-sagittal plane). We estimated the location of subjective straight ahead (SSA) for each subject by using a psychometric fitting function to fit the location judgment results, enabling evaluation of the symmetry of representation between the left and right hemifields. In addition, since the locations of saccadic targets were same in these two tasks, thus, for each individual subject, the elongated saccadic reaction time (SRT) in the latter task, comparing with the former one, mainly reflects the time spent on judgment of the spatial location of visual cue, i.e., spatial perception. We also assessed the efficiency of spatial perception between two hemispheres, through comparing the normalized SRT (i.e., SRT difference between two tasks) between trials with leftward and rightward judgments.

**Results**: Compared with healthy control subjects (HCs), the SSA was shifted to the contralesional side in both left onset PD (LPD, lesion of right substantia nigra) and right onset PD (RPD, lesion of left substantia nigra) patients. The process of spatial information was significantly longer when a spatial cue appeared in the contralesional hemifield.

**Conclusions**: Patients with hemi-PD showed biased visuospatial representation between left and right hemifields and decreased the efficiency of spatial information processing in the contralesional side. Such results indicate that the hemi-PD impairs both spatial representation and the efficiency of spatial information process, which might contribute to asymmetric behaviors.

## Introduction

The classical motor symptoms of Parkinson’s disease (PD) include bradykinesia, muscular rigidity, rest tremor and postural and gait impairment (Kalia and Lang, 2015). In addition, patients with PD commonly exhibit spatially asymmetric behaviors (Lee et al., 2001b; Amick et al., 2006), such as veering while attempting to walk straight ahead (Ren et al., 2015). Spatially asymmetric behaviors in PD were traditionally thought to be entirely caused by motor dysfunction (Lacquaniti et al., 2002; Courtine and Schieppati, 2004). It was recently proposed that spatial perception of PD patients might be also impaired, potentially contributing to spatially asymmetric behaviors (Amick et al., 2007; Davidsdottir et al., 2008; Galna et al., 2012; Kabasakalian et al., 2013; Ren et al., 2015). However, to date, the findings of previous studies examining the spatial perception of PD patients are controversial (Crucian and Okun, 2003; Norton et al., 2015). Thus, whether the spatial perception contributes to spatially asymmetric behavior in PD patients remains unclear.

Measuring the location of subjective straight ahead (SSA) is a well established method for assessing a subject’s representation of external space (Chokron et al., 2004; Kazandjian et al., 2009). Because SSA subjectively separates external space into the left and right halves, a dramatic deviation of SSA indicates unbalanced spatial representation. Many brain diseases have been found to cause SSA deviation (Farnè et al., 1998; Ferber and Karnath, 1999). For example, hemineglect patients were found to exhibit ipsilesional deviation (i.e., contralateral to the neglect hemifield) of the SSA (Richard et al., 2005; Rousseaux et al., 2013), and ipsilesional deviation of SSA has also been reported in PD patients (i.e., contralateral to the onset side of motor-symptom; Davidsdottir et al., 2008). However, because each hemisphere predominantly processes information from the contralateral hemifield, the result of SSA deviating to the ipsilesional side is hard to be interpreted. Conversely, the opposite result (i.e., deviation of the SSA to the contralesional side) is expected, because the intact hemisphere would presumably take over part of the space from the lesional hemisphere. The methodology that used in previous studies might cause such puzzle, because the eye position was not monitored. In fact, eye position might strongly affect the spatial judgment (Morgan, 1978; Barbeito and Simpson, 1991; Lewald and Ehrenstein, 2000). The reason of eye position playing crucial role in spatial judgment is due to that the body-centered reference frames is formed by the integration of information from retinal inputs (vision), eye position and head position signals (Andersen et al., 1997). It has been reported that after lesion of one hemisphere, the eye position was biased to the ipsilesional side (Kato et al., 1995), which might cause ipsilesional deviation of SSA. To solve this puzzle, while we assessed the location of SSA, subjects’ eye position was monitored by an infrared camera eye-tracking system.

In addition to spatial representation, the speed of information processing from sensation to perception is another important aspect of spatial perception, reflecting the efficiency of signal transduction. While previous studies have focused on assessing the features of spatial representation, to our knowledge no study has examined the temporal characteristics of spatial perception in PD patients.

In the present study, we examined two features of spatial perception in hemi-PD patients. First, the location of SSA was measured to assess the symmetry of visuospatial representation. Second, the saccadic reaction time (SRT) was measured to evaluate the efficiency of spatial information processing. We predict that the hemi-PD patients will show the following impairments: the biased SSA deviating toward the ipsilesional side and the increased SRT in the contralesional side due to the asymmetric lesion of dopaminergic system between two hemispheres.

## Materials and Methods

### Participants

A total of 27 right-handed individuals participated in this study: 18 patients (six male, 12 female) with hemi-PD (Hoehn and Yahr stage <2) and nine (four male, five female) age-matched healthy controls (HCs). All participants provided written informed consent. The experimental protocol was approved by the Ethics Committee of the First Clinical College of Harbin Medical University.

The PD patients were divided into right onset PD (RPD; three male, six female) and left onset PD (LPD; three male, six female) based on the onset side of motor symptoms. All patients were diagnosed according to the MDS Clinical Diagnostic Criteria for PD (Postuma et al., 2015). All participants underwent structural magnetic resonance imaging (MRI, 1.5 Tesla) scanning of brain. Patients with the following conditions were excluded from the study: taking anticholinergic drugs, coexistence of serious chronic medical illness, traumatic brain injury, brain surgery or stroke, alcoholism or drug abuse and psychiatric or neurological diseases besides PD. All patients were on their normal medication routine at the time of the experiments: L-dopa (*n* = 1), or pramipexile (*n* = 2), or selegiline (*n* = 4) or combination of above mentioned medicines (*n* = 7). Demographic and clinical data are presented in Table 1.

**Table 1:** Demographic and clinical data of each group.

Subject group	HC	RPD	LPD	*P* value
Sample size	9	9	9	
Men: Women	4:5	3:6	3:6	0.94
Age (years)	61.89 (3.37)	62.44 (5.43)	57.78 (6.76)	0.15
MMSE	29.22 (0.67)	28.67 (1.00)	28.78 (1.30)	0.49
Disease duration (years)	-	2.11 (0.78)	1.78 (0.83)	0.47
UPDRS III	-	13.89 (5.75)	10.78 (3.63)	0.18
H&Y	-	1 (1–1.5)	1 (1–1.5)	0.30
LED (mg)	-	159.72 (154.34)	93.05 (93.36)	0.48

*HC, healthy control; RPD, Parkinson’s disease with motor symptoms initiated on the right side of the body; LPD, Parkinson’s disease with motor symptoms initiated on the left side of the body; MMSE, mini mental state examination; UPDRS, Unified Parkinson’s Disease Rating Scale; H&Y, Hoehn and Yahr stage; LED, levodopa equivalent dosage. Values presented are means (standard deviations) except for H&Y medians (range)*.

All participants completed the Folstein mini mental state examination (MMSE) for screening dementia (MMSE < 26), the Hamilton depression scale examination (HAMD) for screening depression (HAMD > 8) and cancellation tests and copying tasks to screen for visuospatial neglect. All participants had normal or corrected to normal vision. There were no significant differences in age (one-way ANOVA, *F*_(2,24)_ = 2.03, *p* = 0.15), gender (Fisher’s exact test, *p* = 0.94), HAMD scores (one-way ANOVA, *F*_(2,24)_ = 3.08, *p* = 0.06) and MMSE scores (one-way ANOVA, *F*_(2,24)_ = 0.74, *p* = 0.49) among all groups. There were no significant differences between RPD and LPD patients in the duration of disease (Wilcoxon test, *p* = 0.47), Unified PD Rating Scale (UPDRS) III scores (Wilcoxon test, *p* = 0.18), Hoehn and Yahr stage (Kruskal-Wallis test, *p* = 0.30) or levodopa equivalent dosage (LED; Wilcoxon test, *p* = 0.48).

### Apparatus

Subjects were seated in a dark room with their heads restrained on a stable chin rest. All visual stimuli were displayed on a 21-inch CRT monitor (Sony Multiscan G520, 1280 × 960 resolution, 100 Hz refresh rate), positioned 57 cm in front of the subjects. Eye position was monitored using an infrared image eye tracker (Eyelink 1000 desktop mount, SR Research). During the experiments, subjects’ mid-sagittal plane was aligned with the midline of the CRT screen. Stimulus presentation and behavioral data collection was controlled by MATLAB (MathWorks) with Psychtoolbox running on a PC.

To minimize the possible influence of surrounding objects on task performance, all experiments were carried out in an extremely dark environment. The background luminance of the monitor was set to dark. All light-producing components of the apparatus (e.g., the dim light on the keyboard and the power buttons of the computer), were covered with black paper. Thus, during the experiments, subjects could not see the edge of the monitor.

### Behavioral Tasks

#### Location Discrimination Task (LDT, Figure 1A)

**Figure 1:**
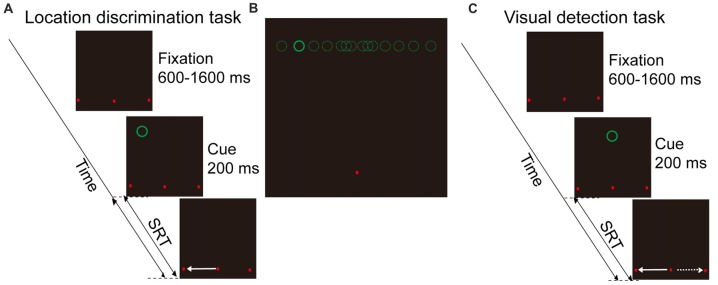
Schematic illustration of behavioral tasks and visual cue locations. **(A)** Location discrimination task (LDT). Subjects need to remain fixation at the fixation point (middle red dot), and make a saccade toward one of the two saccadic targets (left and right red dots) according to the perceived location of cue (green circle, left vs. right hemifield). **(B)** Illustration of the 14 possible locations of visual cue. **(C)** Visual detection task (VDT). The visual display is same as LDT, but there is only one cue location in the body mid-sagittal plane of subject. Subjects are instructed to make saccade either toward the left or right target in a given session.

This is a spatial cue evoked saccade task. The task began with three red dots appearing on the CRT screen. The three red dots were located 9° below the horizontal meridian of the screen in the y-axis dimension, and −18°, 0°, 18° in the x-axis dimension. The middle dot served as the fixation point whereas other two dots were potential saccadic targets. Subjects were instructed to fixate at the middle dot for a random interval (600–1600 ms). A visual stimulus (visual cue; green circle, 1.0° in diameter, luminance: 0.05 cd/m^2^) then appeared for 200 ms in one of 14 potential locations. Subjects were instructed to make a saccadic eye movement as fast as possible toward one of the two potential saccadic targets, according to the perceived location of the visual cue relative to the body mid-sagittal plane. The 14 possible locations of the visual cue were 9° above the horizontal meridian of the screen in the y-axis, and with −15.5°, −10.5°, −6.5°, −3.5°, −1.5°, −1°, −0.5°, 0.5°, 1°, 1.5°, 3.5°, 6.5°, 10.5°, 15.5° in the X-axis (Figure 1B).

#### Visual Detection Task (VDT, Figure 1C)

This is a non-spatial cue evoked saccade task. The time sequence and the locations of two saccadic targets were same as in the location discrimination task (LDT). However, the visual cue appeared only at one location (i.e., on the body mid-sagittal plane). Subjects were instructed to make saccades to one of the targets (always either left or right in a given session) as quickly as possible after the onset of the visual cue. During experiments, subjects were instructed to maintain their gaze at the fixation point before the appearance of a cue. If the eyes moved out of the fixation window (3° radius, centered at the fixation point) during the fixation period, the trial was terminated. In addition, to minimize the possibility that the fixation point would serve as an allocentric reference, we positioned the fixation point 9° below the horizontal meridian, and the cue at 9° above the horizontal meridian. Thus, when the cue appeared around the vertical meridian, it was difficult for subjects to judge the location of a cue based on its spatial relationship with the fixation point.

### Data Analysis and Statistics

We excluded the following trials from data analysis: fixation break trials (HC: 556 out of 6160, 9.03%, RPD: 914 out of 6160, 14.84%, LPD: 667 out of 6160, 10.83%), no response trials (HC: 103 out of 6160, 1.67%, RPD: 201 out of 6160, 3.26%, LPD: 114 out of 6160, 1.85%) and outlier trials (HC: 65 out of 5501, 1.18%, RPD: 48 out of 5045, 0.95%, LPD: 36 out of 5379, 0.67%). No response trials were defined as trials with no saccade, or the amplitude of a saccade was smaller than 4°, within 1000 ms after the cue’s appearance. Outlier trials were defined as trials with SRTs that were differed from the mean value larger than 3 ± SD in each location for each subject. We also excluded a session if the fixation break rate was >30%. In total, 2704 of 18480 trials (14.63%) were excluded.

#### Fitting Function

We employed the cumulative distribution function to fit the data of rightward response:

(1) $$f(x,\mu ,\sigma )=\frac{1}{\sigma \sqrt{2\pi }}\int_{-\infty }^{x}\exp\left(-\frac{{\left(x-\mu \right)}^{2}}{2\sigma^{2}}\right)dx$$

In the cumulative distribution function, *x*, *μ* and *σ* represent cue eccentricity, point of subjective equality (PSE), and skewness of the curve, respectively. The goodness of fitting was examined by *R*^2^. The value was 0.9997, 0.9990, 0.9988 in HC, RPD and LPD, respectively.

#### Definition of SSA

For each subject, we calculated the percentage of rightward responses for each stimulated location (14 locations in total). We then used a psychometric fitting function (cumulative normal distribution) to fit the data. The location of SSA was defined as the corresponding point in the X-axis to the PSE (50% rightward response).

#### SRT Normalization

Since the locations of saccadic targets were same in location discrimination and visual detection tasks (VDTs), the SRT difference between the two tasks under conditions that saccades directed to the same target reflected the difference in the process of spatial perception and visuomotor transformation but not in saccadic control. Thus, we first calculated the normalized SRT for each subject before performing further statistical analysis. The detail methods to calculate normalized SRT are described in bellow.

(2) $$nSRT_{\left(\text{dir}\right)}=SRT_{\left(\text{dir,LDT}\right)}-SRT_{\left(\text{dir,VDT}\right)}$$

nSRT_(dir)_ represents the normalized SRT in a given direction; SRT_(dir, LDT)_ represents the mean SRT in the same direction in LDT; SRT_(dir, VDT)_ represents the mean SRT in the same direction in VDT.

## Results

### Significant Shift of SSA in PD Patients Compared With HC Subjects

**Figure 2:**
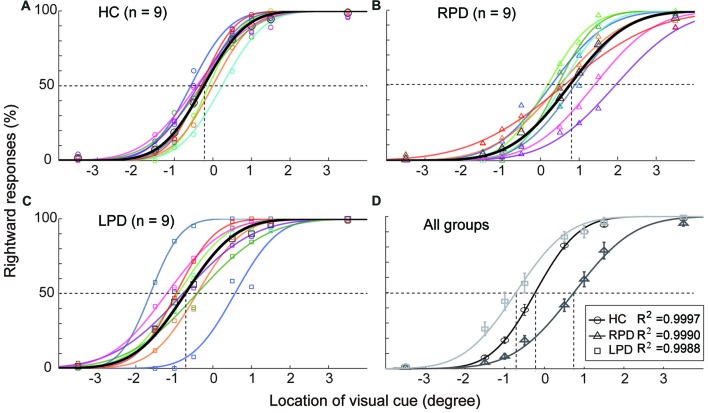
The opposite shift of subjective straight ahead (SSA) between right onset PD (RPD) and left onset PD (LPD). **(A–C)** The distribution of rightward judgment as a function of cue location in healthy control (HC), RPD and LPD. Data were fitted by a psychometric model. **(D)** The comparison of SSA among three groups of subjects. The location of SSA was defined as the corresponding point in X-axis to the point of subject equality (point of subjective equality (PSE), 50% rightward response). Positive values represent right hemifield, and negative values represent left hemifield.

Each subject’s SSA was evaluated by fitting the data of location judgment for each visual cue location with a cumulative distribution function (Figure 2).The location of the SSA was defined as the corresponding point on the X-axis to the PSE (50% rightward response).

The results of the HC group (Figure 2A) showed that the SSA in eight of nine subjects slightly deviated to the left hemifield (mean = −0.23°, SEM = 0.08) relative to the body mid-sagittal plane (*p* = 0.002, Wilcoxon test). In contrast, the SSA of all RPD patients (Figure 2B) was shifted to the right hemifield (mean = 0.72°, SEM = 0.19), whereas, in eight of nine LPD subjects (Figure 2C), SSA was shifted to the left hemifield (mean = −0.72°, SEM = 0.20). Compared with the HC group, the average SSA of both the RPD and LPD groups were significantly shifted (Wilcoxon test, RPD, *p* < 0.001; LPD, *p* = 0.01), but in the opposite direction (Figure 2D). The Bonferroni correction of Alpha level is 0.025 (α’= 0.05/2).

### The Opposite Deviation of SSA Between Left and Right Hemi-PD Patients Was Not Correlated With the Eye Position

**Figure 3:**
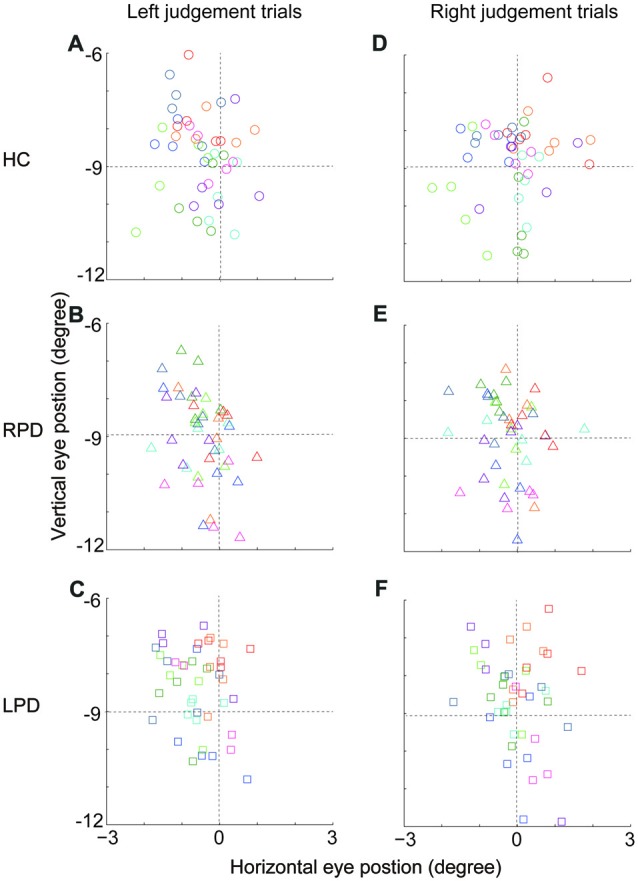
The comparison of eye position across HC, RPD and LPD. **(A–C)** The average eye position (from visual cue onset to 30 ms before saccade onset) of left judgment trials in three groups of subjects, respectively. Eye position data were analyzed when visual cue appeared at the location that was nearest to the SSA (HC, −0.5°; RPD, 0.5°; LPD −1.0°). **(D–F)** The average eye position of right judgment trials. Each symbol presents the average eye position in a session. Same colored symbols represent data of an individual subject. Positive values represent right hemifield, and negative values represent left hemifield.

Since eye position can strongly affect the spatial judgment (Morgan, 1978; Jeannerod and Biguer, 1989; Barbeito and Simpson, 1991; Lewald and Ehrenstein, 2000), it is possible that the opposite SSA deviation between RPD and LPD was related to the deviation of eye position during location judgment of the visual cue, even though subjects were required to maintain fixation straight ahead. To examine this possibility, we calculated the eye position of each subject while the visual cue appeared at the location that was nearest to the SSA (HC, −0.5°; RPD, 0.5°; LPD −1.0°), and compared the difference in eye positions among subjects of three groups (HC, LPD and RPD) when they made same directional judgment. The averaged eye positions (from visual cue onset to 30 ms before saccade onset) in each session for each subject are shown in Figure 3. For each subject, trials were separated into two groups based on the location judgment of the visual cue (i.e., left (Figures 3A–C) vs. right (Figures 3D–F)) judgment. One-way ANOVA revealed no significant difference in eye position (in the horizontal dimension) among HC, RPD and LPD in both left (*F*_(2,129)_ = 0.46, *p* = 0.66) and right judgment (*F*_(2,129)_ = 0.75, *p* = 0.48). These results indicated that the opposite shift of SSA between RPD and LPD was not correlated with eye position when subjects made a location judgment about the visual cue.

### There Was No Systematic SRT Difference Between Leftward and Rightward Saccades in the Visual Detection Task Within Group and Between Groups of Subjects

**Figure 4:**
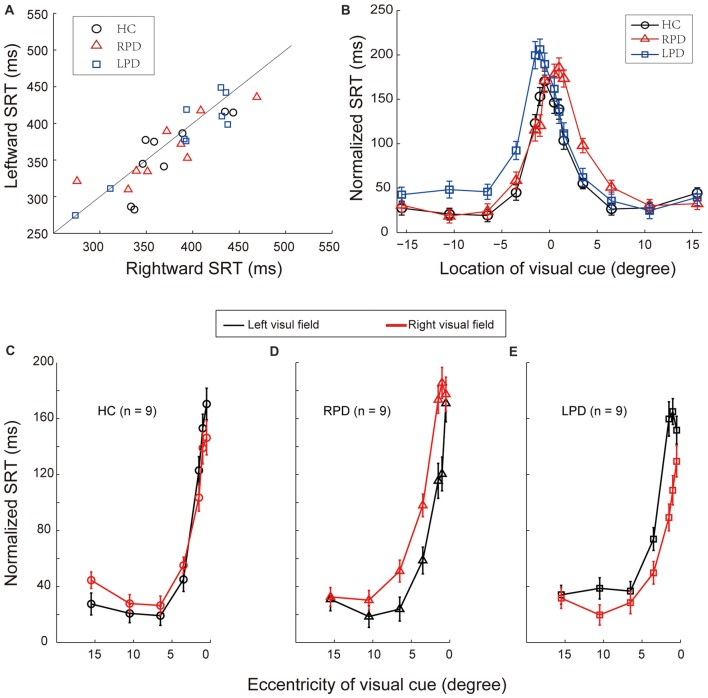
The comparison of saccadic reaction time (SRT) in VDT and normalized SRT in LDT. **(A)** The comparison of SRT of each individual subject in the VDT. **(B)** The comparison of the distribution of normalized SRT in the LDT among HC, RPD and LPD. Positive values represent right hemifield, and negative values represent left hemifield. **(C–E)** Normalized SRT in LDT in HC, RPD and LPD, respectively. Red symbols represent data of right hemifield, black symbols represent data of left hemifield.

We examined whether there was a systematic difference in SRT between leftward and rightward saccades in the simple visually evoked saccades, i.e., the VDT. To do so, we directly compared the average SRTs of two opposite saccades for each individual subject (Table 2, Figure 4A). The analysis of population SRT data by two-way repeated-measures ANOVA (groups and saccade directions) showed no systematic differences in SRTs within group (*F*_(1,24)_ = 3.16, *p* = 0.09) and among groups (*F*_(2,24)_ = 0.63, *p* = 0.54). In addition, there was no significant interaction between the two factors (*F*_(2,24)_ = 0.45, *p* = 0.64). Such results indicate that, in hemi-PD patients, the basic process of visual-oculomotor transformation underlying simple visually-guided saccades remains normal.

**Table 2:** Saccadic reaction time (SRT) of each group in the visual detection task (VDT; ms).

	HC	RPD	LPD
Leftward	358.31 (49.19)	363.08 (43.77)	384.27 (58.21)
Rightward	373.38 (40.93)	369.71 (54.77)	388.54 (58.77)

*Values presented are means (standard deviations)*.

### The Process of Spatial Information Took Longer in PD Patients When Visual Cues Appeared in the Contralesional Hemifield

Since SRT reflects the time from visual cue onset to saccade initiation, it contains the following processes: visual perception, visual-oculomotor transformation and saccadic control. Thus, to assess the temporal characteristics of spatial information processing, we need to subtract the time spent in saccadic control from SRT. To do so, we calculated the SRT difference, named as normalized SRT, between the LDT and VDT when saccades directed to a same target location. The normalized SRT reflects the time spent in spatial information processing in the LDT. Therefore, the difference in normalized SRTs between two opposite saccades reflects the time difference in spatial information processing between two hemispheres.

The distributions of average normalized SRTs of the three groups of subjects are shown in Figure 4B. The compare of normalized SRTs between left and right hemisphere in each group with one-way repeated-measures ANOVA. The results were as following (Figures 4C–E): the normalized SRT was significantly different between left and right hemifields in RPD (*F*_(1,86)_ = 8.12, *p* = 0.005) and LPD (*F*_(1,86)_ = 7.23, *p* = 0.009) patients, but not in HC (*F*_(1,86)_ = 0.06, *p* = 0.80). Compared with HC, there was a significant difference in normalized SRT in the contralesional hemifield in PD patients (RPD, *F*_(1,86)_ = 10.55, *p* = 0.002; LPD, *F*_(1,86)_ = 11.35, *p* = 0.001), but not in the ipsilesional hemifield (RPD, *F*_(1,86)_ = 0.07, *p* = 0.79; LPD, *F*_(1,86)_ = 0.12, *p* = 0.73). The Bonferroni correction of Alpha level is 0.025 (α’= 0.05/2).

Such results indicate that the efficiency of spatial information processing in PD is reduced in the contralesional side.

## Discussion

The neurodegeneration of PD in early stage is commonly asymmetric in the nigrostriatal pathways between the two hemispheres (Rinne et al., 1993; Wang et al., 2015). LPD results from the predominant dysfunction in the right hemisphere, and vice versa for RPD (Verreyt et al., 2011; Lee et al., 2015). In the present study, we assessed the visual spatial representation as well as the efficiency of spatial information processing in hemi-PD patients. Patients were divided into two groups based on the onset side of motor symptoms: left onset motor-symptoms PD (LPD, lesion of right substantia nigra) and right onset motor-symptoms PD (RPD, lesion of left substantia nigra). Compared with HC, hemi-PD patients exhibited significant deviation of SSA to the contralesional hemifield (e.g., the side of onset of motor-symptoms) in both LPD and RPD patients, and SRT was significantly longer when saccades were directed to the contralesional hemifield. Thus, the SSA and SRT results indicated that both the spatial representation and efficiency of spatial information processing are impaired in the contralesional hemifield of early PD patients.

To assess the spatial features of spatial perception, we measured the location of the visual SSA, which subjectively separated the external space into the left and right halves (Jeannerod and Biguer, 1989). The SSA data revealed that, compared with HC, the SSA of hemi-PD patients significantly shifted toward the contralesional hemifield, such that LPD shifted to left hemifield and RPD shifted to right hemifield (Figure 2). These results suggest that the spatial representation of contralesional space in hemi-PD patients may have been shrunk or compressed, consistent with the findings of previous studies (Lee et al., 2001a; Harris et al., 2003).

In contrast to the current results, Davidsdottir et al. (2008) reported an ipsilesional shift (i.e., contralateral to the onset side of motor-symptom) of SSA in cortical and subcortical lesions. Although there are clear differences in the behavioral paradigms used in these studies, the effect of eye position on spatial judgment may have also played an important role in the conflicting findings (Jeannerod and Biguer, 1989). It has been previously reported that, fixation points shifted to the ipsilesional side during free viewing, and saccadic eye movements occurred more often in the ipsilesional hemifield (Kato et al., 1995). The bias of eye position toward the ipsilesional side might cause the judgment of SSA shift to the ipsilesional side, because the retinotopic reference frame affects spatial perception in head/body centered reference frames (Jeannerod and Biguer, 1989). However, because previous studies did not monitor eye position, as in the current study, it is difficult to directly compare the current findings with previous reports.

To assess the temporal features of spatial perception, we measured SRT, which reflects the time of signal transduction from visual cue onset to saccade initiation. SRT serves as a behavioral probe that indicates the efficiency of information processing in neural circuits. However, because the signal processing from perception and sensorimotor transformation to action is partially overlapping and continuous, it is extremely difficult to dissociate them from each other. Thus, while the spatial features of perception (e.g., location discrimination and external space representation) have been extensively studied (Pizzamiglio et al., 2000; Chokron et al., 2004; Kazandjian et al., 2009), the temporal features of perception have rarely been explored. Here, we designed two saccadic tasks: VDT and LDT. Importantly, the two tasks require subjects to make same saccade toward either the left or right saccadic target. In other words, the motor control for saccades to a given target are the same between the two tasks. Thus, for an individual subject, the difference in saccadic control between two opposite saccades should be the same in both tasks.

We calculated the SRT difference, named as normalized SRT, between the LDT and VDT when saccades directed to a same target location. The normalized SRT reflects the time spent in spatial information processing in the LDT. Therefore, the difference in normalized SRTs between two opposite saccades reflects the time difference in spatial information processing between two hemispheres. Results indicate that spatial information processing was slower in the contralesional hemifield of hemi-PD patients (Figure 4B).

Finally, our SRT data revealed that, in the simple task condition (VDT), the efficiency (speed) of visual-oculomotor transformation between left and right hemifields in PD patients was similar, but in the complex task condition (LDT) it was significantly reduced in the contralesional hemifield. These results are consistent with the findings of previous studies (Mosimann et al., 2005), supporting the notion that spatial perception is impaired in PD.

In summary, our SSA and SRT results indicate that both the spatial representation and efficiency of spatial information processing are impaired in the contralesional hemifield of early PD patients. The possible underlying neural mechanisms are that, the dysfunction of basal ganglia-thalamus-cortex circuitry in PD extends to the prefrontal and posterior parietal lobes (Middleton and Strick, 2000; Bartels et al., 2006), the crucial structure for spatial perception, thus PD might impair not only the motor function but also the spatial perception (Amick et al., 2007; Davidsdottir et al., 2008; Galna et al., 2012; Kabasakalian et al., 2013; Ren et al., 2015).

## Author Contributions

DS, YZ and YP initiated and designed the experiments. DS collected data with the assistance of LL, LZ and ML. DS, ML and WZ analyzed the data. DS, YP and MZ wrote the manuscript.

## Conflict of Interest Statement

The authors declare that the research was conducted in the absence of any commercial or financial relationships that could be construed as a potential conflict of interest.

## Abbreviations

LDT: Location discrimination task
LPD: left onset PD
PD: Parkinson’s disease
PSE: point of subjective equality
RPD: right onset PD
SRT: saccadic reaction time
SSA: subjective straight ahead
VDT: Visual detection task
VF: visual field.
